# Time-to-positivity of *Mycobacterium avium* complex in broth culture associates with culture conversion

**DOI:** 10.1186/s12879-022-07250-4

**Published:** 2022-03-12

**Authors:** Christina M. Mingora, Bryan A. Garcia, Kevin C. Mange, Dayton W. Yuen, Monika Ciesielska, Jakko van Ingen, Patrick A. Flume, Susan E. Dorman

**Affiliations:** 1 Medical University of South Carolina, Charleston, SC USA; 2 University of Alabama at Birmingham, Birmingham, AL USA; 3 Insmed Incorporated, Bridgewater, NJ USA; 4 Radboudumc Center for Infectious Diseases, Department of Medical Microbiology, Radboud University Medical Center, Nijmegen, The Netherlands; 5 Medical University of South Carolina, 135 Rutledge Avenue, Room 1207, Charleston, SC 29425 USA

**Keywords:** Non-tuberculous mycobacteria, *Mycobacterium avium* complex, Biomarker

## Abstract

**Background:**

Mycobacterial time to positivity (TTP) in liquid culture media has predictive value for longer term outcomes in pulmonary tuberculosis, but has not been thoroughly studied in nontuberculous mycobacterial pulmonary disease. This study sought to evaluate for association between TTP and sputum culture conversion to negative in pulmonary disease caused by *Mycobacterium avium* complex (MAC).

**Methods:**

Data from the CONVERT trial (NCT02344004) that evaluated efficacy of guideline-based-therapy with or without amikacin liposome inhalation suspension in adults with refractory MAC-PD (*Mycobacterium avium* complex pulmonary disease) were analyzed. We evaluated TTP measures for sputum obtained prior to study treatment initiation and at monthly visits, assessing reproducibility of measures as well as association of TTP with culture conversion on treatment.

**Results:**

Data from 71 participants with at least one screening visit TTP value were analyzed. For participants who provided more than one sputum sample at a given visit, there was moderate between-sample reliability, with median intraclass correlation coefficient 0.62 (IQR 0.50, 0.70). Median TTP at screening was longer in those participants who subsequently achieved vs. did not achieve culture conversion (10.5 [IQR 9.4] days vs. 4.2 [IQR 2.8] days, p = 0.0002). Individuals with culture conversion by study treatment month 6 were more likely to have a screening TTP > 5 days compared to those who did not achieve culture conversion (OR 15.4, 95% CI 1.9, 716.7, p = 0.0037) and had increasing TTPs over time.

**Conclusions:**

TTP prior to and on treatment is associated with microbiological treatment response in patients with MAC-PD.

**Supplementary Information:**

The online version contains supplementary material available at 10.1186/s12879-022-07250-4.

## Background

The prevalence of non-tuberculous mycobacterial pulmonary disease (NTM-PD) is increasing, and species from the *Mycobacterium avium* complex (MAC) are the most common etiology [1–3]. Current guidelines for treatment of MAC pulmonary disease (MAC-PD) recommend a prolonged course of combination antimicrobial therapy [4]. Despite treatment, outcomes remain sub-optimal and conversion of sputum cultures to negative, considered a surrogate for pathogen eradication, is not universally achieved; a meta-analysis reported a sputum culture conversion rate of 60% and a recent expanded systematic review reported a culture conversion rate of 56.5% [5–7]. In addition, treatment is often associated with patient intolerance and systemic toxicities [4]. Therefore, there is a need for development of novel therapeutics with both improved efficacy and tolerability.

Currently, there are no reliable biomarkers that predict response to therapy for NTM-PD, leaving clinicians to rely heavily on results from respiratory cultures as an objective measure of clinical response. However, reliance on respiratory culture conversion is problematic because this outcome takes months to achieve, thereby delaying assessment of a patient’s response to treatment and presenting a barrier for clinical trials. This gap in knowledge highlights the critical need for an early marker of microbiological efficacy against NTM. For pulmonary tuberculosis, time-to-positivity (TTP) of *Mycobacterium tuberculosis* in liquid culture media correlates well with conventional colony forming unit (cfu) counting, pre-treatment TTP inversely correlates with treatment response, and change in TTP over time on treatment is a biomarker of therapeutic response [8–10]. For MAC-PD, TTP correlates well with conventional sputum cfu counting, prompting new research to investigate correlation with treatment outcomes [11].

We sought to assess the relationship between TTP and microbiologic response to treatment of MAC-PD using existing data collected in the CONVERT trial (NCT02344004), which evaluated the addition of amikacin liposome inhalation suspension to guideline-based therapy in patients with refractory MAC-PD [12]. Participants in the CONVERT trial provided sputum specimens at pre-specified intervals prior to and during study treatment, with culture conversion to negative as the primary trial outcome, thus providing an opportune sample for our secondary investigation. Preliminary results of our study have been previously reported in the form of an abstract [13].

## Methods

Prior to enrollment and participation in the CONVERT trial, informed consent was obtained from all subjects and/or their legal guardians. Methodology of the initial Phase III clinical trial is described and summarized below. Our secondary data analysis, also described below, was determined to be exempt human subjects research, under Exemption 4, by the IRB of the Medical University of South Carolina. All methods described were carried out in accordance with relevant guidelines and regulations.

### CONVERT trial

The trial enrolled adults with treatment-refractory MAC-PD who had MAC positive sputum or bronchoscopy cultures at the time of study eligibility screening. Baseline was defined as the interval of time from 2 days prior to initiation of study treatment to the day of study treatment start. The CONVERT primary endpoint was the proportion of participants achieving culture conversion to negative, based on cultures from sputum obtained monthly from baseline through month 6 and considering the results of liquid and solid media cultures. For a given participant, culture conversion was considered to be achieved if there were 3 consecutive monthly negative sputum cultures, with all sputum samples collected at each visit required to be culture-negative. To meet the primary endpoint, month 4 was the latest visit at which a negative sputum culture could first be detected. The protocol specified that at least two and preferably three sputum specimens were to be obtained from each participant at each assessment. Each sputum specimen was collected on consecutive days prior to a scheduled visit, except for screening specimens, which were collected during the week following the screening visit. If a participant was not able to spontaneously expectorate, sputum induction was attempted once at the study visit. Participants were instructed to refrain from administration of the inhaled study treatment on the days that sputum was obtained, starting 2 days prior to each scheduled visit. Sputum specimens were refrigerated until shipped to a designated study laboratory within 2 days of collection.

In the laboratory, sputum was digested and decontaminated using *N*-acetyl-l-cysteine/sodium hydroxide (final concentration of sodium hydroxide 1%). After centrifugation, the sediment was resuspended in phosphate buffered saline and cultured on Lowenstein–Jensen solid agar and in Mycobacteria Growth Indicator Tubes (MGIT, Becton, Dickinson & Company, Franklin Lakes, NJ) using the MGIT 960 system, which automatically flags positive cultures and calculates the TTP. MGIT inoculation volume was 0.5 mL, and MGIT cultures were incubated for 6 weeks before being reported as negative. Mycobacteria were identified using a commercial DNA probe assay that includes a general MAC probe as well as species-specific probes for *M. avium*, *M. intracellulare* and *M. chimaera* (InnoLiPA Mycobacteria v2, Innogenetics, Ghent, Belgium).

### Secondary TTP data analysis

Summaries were performed on existing data from the CONVERT trial using the subset of the intention to treat analysis population with available TTP data, rather than an a priori design with powering for the comparisons of interest. Confidence intervals (CIs) and corresponding p-values should be regarded as nominal. All analyses were performed on available data without imputations. Baseline demographics were summarized using descriptive statistics. The intraclass correlation coefficient (ICC) was used to describe between-sample reliability for those participants who provided more than one sample at a given visit and was derived using random effect coefficient modeling. Bland–Altman methodology was used to assess TTP reproducibility between screening and baseline visits, a period during which each participant’s treatment was held constant. Comparison of TTP between sputum culture converters and non-converters was performed via logistic regression. Comparative analyses were not adjusted for multiplicity; hence p-values are nominal.

## Results

### Participants

The CONVERT study used three reference laboratories for microbiological testing using broth culture as per CONVERT study protocol, however precise to the hour TTP data were available only from the Radboudumc Center for Infectious Diseases laboratory, the Netherlands. Thus, although the CONVERT study enrolled 336 participants overall, only 71 participants had at least one screening TTP value and were included in the current analysis. Baseline demographics, treatment assignment, and culture conversion rates for this subset of participants as well as those included in the CONVERT intention to treat analysis population are shown in Table 1. Compared to the larger CONVERT population, our sample included a higher proportion of Caucasians, lower proportion with culture conversion, and variability in underlying lung pathology, but otherwise was similar.

**Table 1:** Characteristics of study participants in the CONVERT trial and in the time to positivity secondary analysis

Characteristic	Intention-to-treat analysis population in CONVERT trial (N = 336)	At least one screening time to positivity value (N = 71)
Age in years, mean (SD)	64.7 (9.77)	65.6 (8.62)
Race: white, n (%)	235 (69.9)	62 (87.3)
Sex: female, n (%)	233 (69.3)	45 (63.4)
Baseline body mass index (kg/m^2^), mean (SD)	21.17 (3.875)	21.23 (3.870)
Tobacco use: current smoker, n (%)	36 (10.7)	13 (18.3)
Lung disease, n (%)		
COPD	48 (14.3)	24 (33.8)
Bronchiectasis	211 (62.8)	26 (36.6)
COPD and bronchiectasis	40 (11.9)	11 (15.5)
Duration of NTM disease in years, mean (SD)	5.7 (5.07)	4.9 (4.95)
Duration of NTM treatment prior to study enrollment in years, mean (SD)	3.9 (3.9)	3.9 (4.0)
Assigned treatment in CONVERT trial		
ALIS plus guideline-based therapy, n (%)	224 (66.7)	43 (60.6)
Guideline-based therapy only, n (%)	112 (33.3)	28 (39.4)
Conversion of sputum cultures to negative by month 6, n (%)	75 (22.3)	10 (14.1)

SD, standard deviation; COPD, chronic obstructive pulmonary disease; NTM, nontuberculous mycobacterial disease; ALIS, amikacin liposomal inhalation suspension

### Within-participant reproducibility of TTP measures

**Fig. 1:**
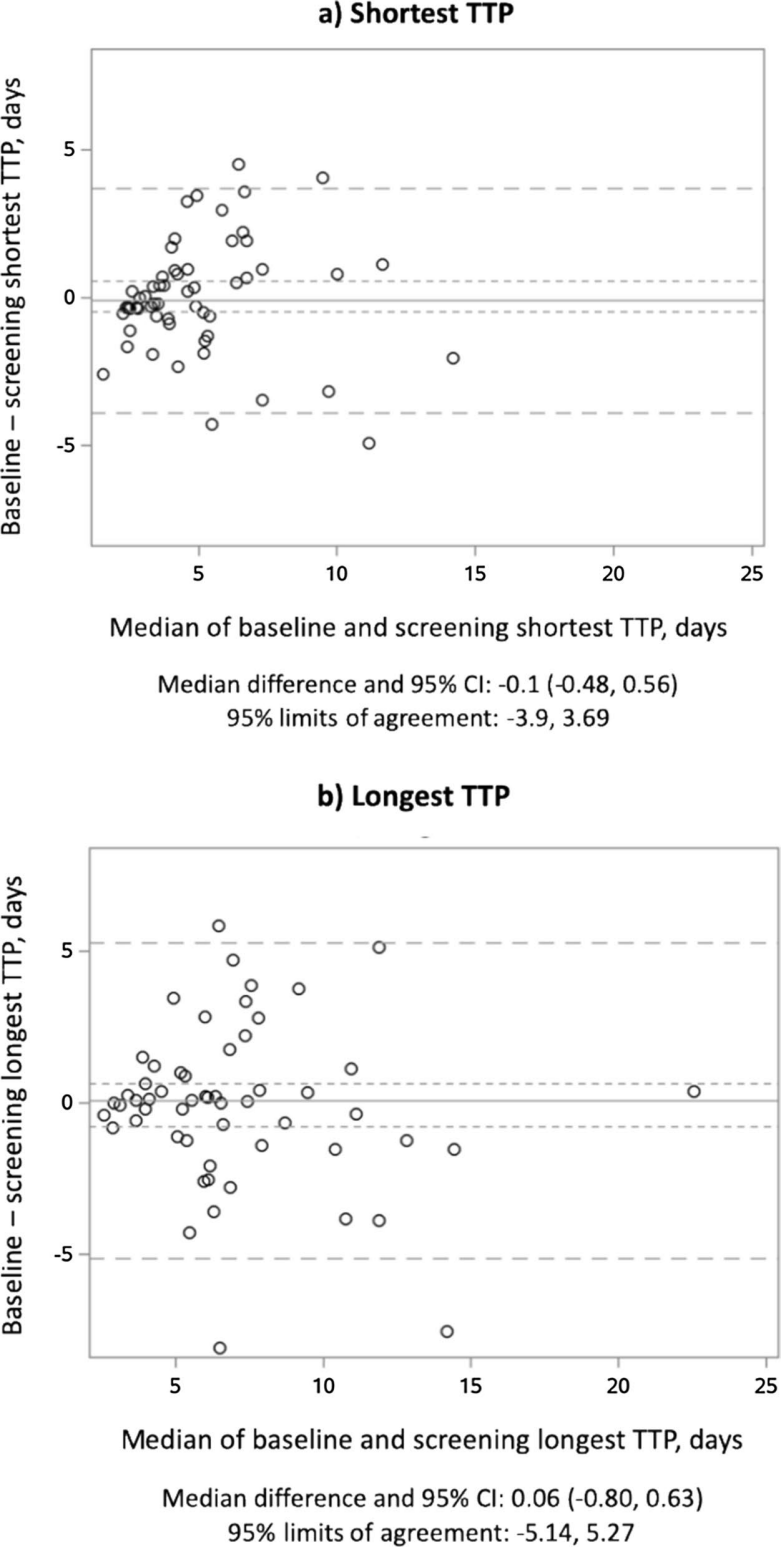
Reproducibility between screening and baseline time to positivity of *Mycobacterium avium* complex in liquid culture. Bland–Altman scatter diagrams of the differences between baseline visit time to positivity (TTP) and screening visit TTP plotted against the median of the two measures for the shortest TTP values (**a**) and the longest TTP values (**b**). The solid line shows the median and the short horizontal dashed lines represent the 95% confidence intervals of the median. Long horizontal dashed lines represent the 95% limits of agreement, defined as the median difference plus and minus 1.96 times the standard deviation of the differences

TTP data were available for 945 sputum cultures obtained at a total of 414 study visits. For those 414 visits, 99 (24%) had one evaluable sputum specimen, 99 (24%) had two evaluable sputum specimens, and 216 (52%) had three evaluable sputum specimens (Additional file 1: Table S1). For those participants who provided more than one sample at a given visit, there was moderate between-sample reliability, with intraclass correlation coefficient (ICC) ranging between 0.39 and 0.74 (median 0.62, IQR 0.50, 0.70, Additional file 1: Table S2). Bland–Altman analysis of TTP reproducibility between screening and baseline visits showed median difference of − 0.1 days (95% CI − 0.48, 0.56) with 95% limits of agreement − 3.9, 3.69 days when individuals’ shortest TTP values were used, and median difference of 0.06 days (95% CI − 0.80, 0.63) with 95% limits of agreement − 5.14, 5.27 days when individuals’ longest TTP values were used (Fig. 1).

### Association of screening TTP with culture conversion

The median TTP at screening was longer in those participants who eventually achieved culture conversion compared to those who did not: 10.5 (IQR 9.4) days vs. 4.2 (IQR 2.8) days, p = 0.0002 when the shortest measured screening TTP was used for each participant; and 10.9 (IQR 7.0) days vs. 6.3 (IQR 4.3) days, p = 0.0013 (Table 2) when the longest measured TTP was used for each participant.

**Table 2:** Summary statistics of screening time to positivity in days, by sputum culture conversion status

Time to positivity value analyzed	Converter status	N	Median	Inter-quartile range	Range	Mean	Standard deviation
Shortest	Any	71	4.5	3.6	2.1, 24.6	6.0	4.1
	Yes	10	10.5*	9.4	4.5, 24.6	11.2	6.4
	No	61	4.2*	2.8	2.1, 13.8	5.2	2.9
Longest	Any	71	7.0	4.8	2.5, 24.6	7.9	4.6
	Yes	10	10.9^†^	7.0	5.8, 24.6	12.1	5.6
	No	61	6.3^†^	4.3	2.5, 22.4	7.2	4.1

*p = 0.0002 for comparison of median shortest TTP for the group with vs. without culture conversion

^†^p = 0.0013 for comparison of median longest TTP for the group with vs. without culture conversion

**Fig. 2:**
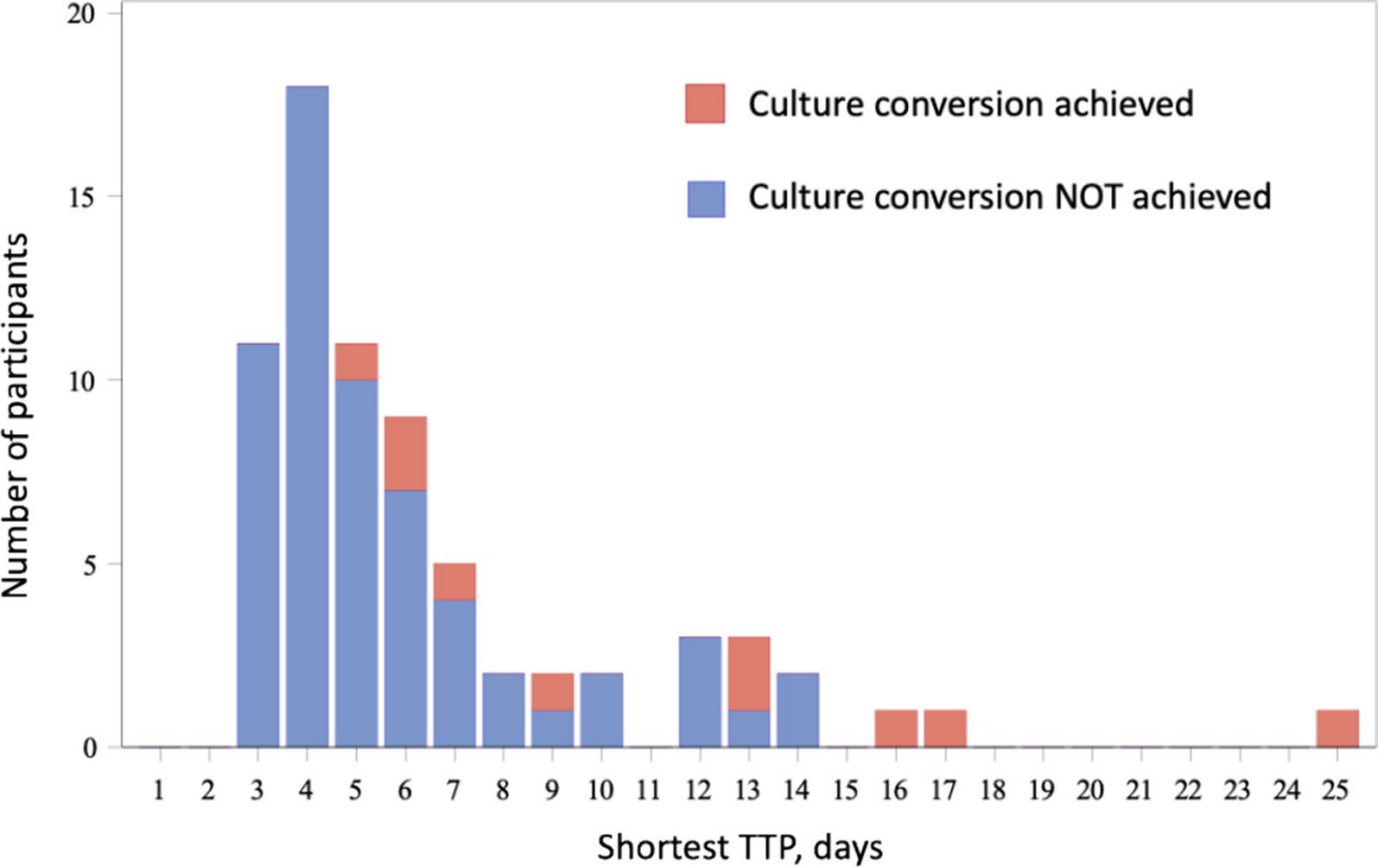
Distribution of shortest time to positivity measured at screening, by converter status. Distribution of screening time to positivity (TTP) by culture conversion status. Red bars represent participants who subsequently achieved culture conversion to negative by month 6 of study participation, and blue bars represent participants who did not achieve culture conversion. TTP values are rounded up to the next whole day; for participants with more than one screening TTP value, the shortest TTP value was used

Figure 2 shows the shortest TTP measured at screening for each participant, demonstrating that all participants who subsequently achieved culture conversion had a screening TTP of at least 5 days or more. Individuals with sputum culture conversion to negative by study treatment month 6 were significantly more likely to have a screening TTP > 5 days than those who did not achieve culture conversion (OR 15.4, 95% CI 1.9, 716.7; p = 0.0037); using a screening TTP threshold of > 7 days yielded OR 6.6, 95% CI 1.3, 37.5; p = 0.0192.

### Association of TTP change over time with culture conversion

**Fig. 3:**
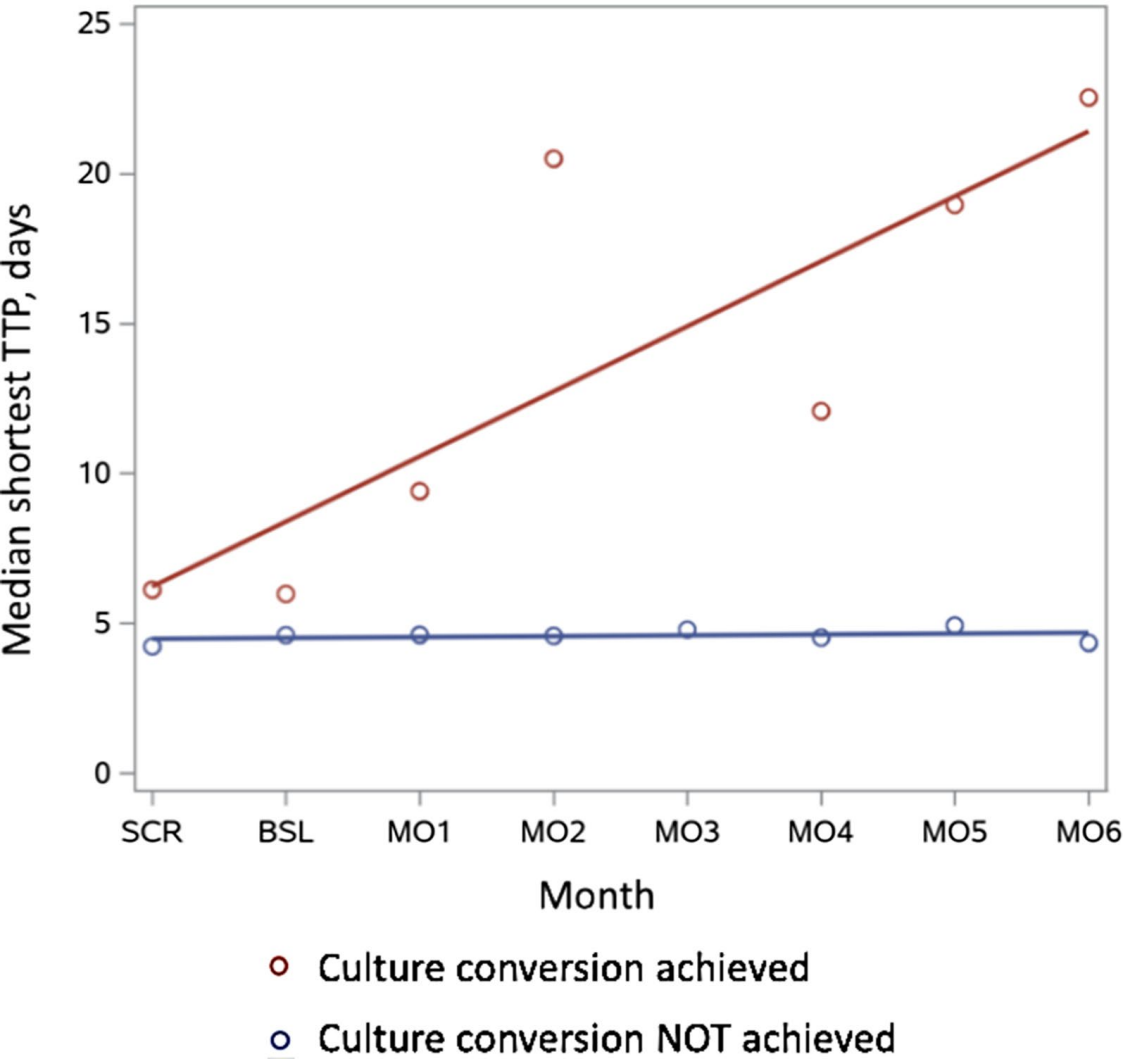
Time to positivity over time, by converter status. Regression analysis of shortest time to positivity (TTP) over time for converters (red) vs. non-converters (blue). *SCR* screening, *BSL* baseline

Figure 3 and Additional file 1: Table S3 show the median TTPs measured at all study visits, stratified by participant culture conversion status. Among participants who did not achieve culture conversion the median TTPs ranged from 4.2 to 4.9 days, without appreciable change over time. Among participants who achieved culture conversion the median TTPs ranged from 10.5 to 22.5 days and increased over time.

## Discussion

Our study extends the current evidence base for TTP as a measure of MAC bacillary burden in two main ways [11]. First, there was a significant inverse association between screening TTP and sputum culture conversion to negative in adults with refractory MAC-PD. This aligns well with clinical observations that individuals with cavitary MAC-PD, which can be accompanied by high bacterial loads reflected by positive sputum smears, have poorer outcomes than those with noncavitary MAC-PD [14]. Second, TTP increased over time in individuals with eventual culture conversion but not in those without culture conversion, and supports exploration of the use of longitudinal TTP measures for treatment response monitoring. Analogous findings have been described for pulmonary tuberculosis—in the laboratory setting TTP correlates with the size of a bacterial inoculum, and in the clinical setting TTP can be used as an alternative to cumbersome colony forming unit quantitation in evaluating response to tuberculosis treatment, both at the individual patient level and as a clinical trial outcome measure [9, 10, 15].

Our results confirm and extend the results of retrospective studies reported recently by others who have investigated the role of TTP in MAC-PD [16, 17]. As in our study, Danho et al. observed longer baseline TTPs in patients who subsequently had versus did not have conversion of cultures to negative, and TTP increased over the 1st months of treatment in individuals who converted cultures to negative. Our study provides useful new information about the within-participant reproducibility of MAC TTP measurements across sputum specimens obtained over 2 to 3 days around each study visit as well as over a longer interval of time in the absence of a change in treatment (i.e. as measured at screening and baseline visits). Reproducibility of the measure was moderate to good, but not perfect, and probably reflects several factors including intensity of cough and/or participant effort, diurnal variation in cough and/or sputum production, and airways whose contents are represented in the expectorated sputum specimen. At the individual participant level, TTP variability between the screening and baseline visits was slightly less when the shortest, rather than the longest, TTPs were analyzed. For this reason and because the shorter TTP is likely to reflect the greater bacterial burden, we elected to use the shorter TTP in our analyses.

Identification of biomarkers for NTM treatment response has been identified as a research priority both by clinicians/investigators and individuals with NTM-PD [18]. TTP is operationally attractive because the TTP value is automatically calculated and reported by MGIT system instruments; while clinical results reporting algorithms may require adjustment to incorporate TTP results, no new technology or instrumentation is needed. The identification of association between TTP at screening and subsequent on-treatment culture conversion has implications for clinical trial design. Consideration should be given to stratified randomization based on pre-intervention bacillary burden as assessed by TTP in order to ensure balance of treatment groups with respect to this prognostic variable.

There are important limitations of our study. Our analyses included only the subset of trial participants for whom TTP data were available. Consequently, the sample size was relatively small overall and especially with regard to participants who achieved culture conversion. Disease was assumed to be stable in the time between screening and baseline assessment, however no detailed objective data was collected to fully support this assumption. We were also unable to explore potential associations between TTP and radiographic features or other clinical severity indices, since this information was not captured systematically during the phase 3 trial. Outcome was expressed as culture conversion status by month 6 of study treatment in order to align with the primary outcome of the CONVERT trial. While our analysis demonstrated a clear signal supporting clinical utility of TTP, larger prospective studies that explore other outcomes and systematically gather detailed baseline severity data may be able to rigorously identify nuanced relationships between TTP and other microbiological or clinical endpoints.

## Conclusions

Our study population, reflecting the CONVERT trial study population, had refractory MAC-PD with persistently positive cultures despite long-term antibiotic therapy prior to study entry whereas tuberculosis studies incorporating TTP have been performed in treatment-naïve individuals. This is a major methodologic difference and antimicrobial treatment may impact mycobacterial TTP through multiple mechanisms. However, CONVERT participants were representative of an important large subset of NTM-PD patients in which a predictive biomarker is particularly needed. Our promising findings in a clinically complex patient population highlight the importance of further investigation of TTP in MAC-PD, including in treatment naïve populations.

While our findings are not conclusive, they are supportive that TTP may serve as a useful biomarker of treatment response in patients with MAC-PD. Our findings provide rationale for incorporation of TTP measurements into clinical trials of novel therapies for MAC-PD and into clinical practice.

## Supplementary Information

**Additional file 1: Table S1.** Summary of time to positivity (TTP) counts, by visit. **Table S2. **Repeatability of time to positivity, by visit. **Table S3.** Summary statistics of shortest time to positivity, in days, by visit for participants with at least one screening time to positivity value.

## Data Availability

All data generated or analyzed during this study are included in this published article [and its additional information files].

## Abbreviations

TTP: Time to positivity
MAC: *Mycobacterium avium* Complex
MAC-PD: *Mycobacterium avium* Complex pulmonary disease
NTM: Non-tuberculous mycobacteria
NTM-PD: Non-tuberculous mycobacterial pulmonary disease
CFU: Colony forming unit
MGIT: Mycobacteria growth indicator tube
CI: Confidence interval
ICC: Intraclass coefficient
IQR: Interquartile range
OR: Odds ratio
