# Supplementary material for: Time-to-positivity of Mycobacterium avium complex in broth culture associates with culture conversion
Source: BMC Infect Dis. 2022 Mar 12;22:246. doi: 10.1186/s12879-022-07250-4 (PMC8918293; doi:10.1186/s12879-022-07250-4)
Supplement: Supplementary file 1 — Additional file 1: Table S1. Summary of time to positivity (TTP) counts, by visit. Table S2. Repeatability of time to positivity, by visit. Table S3. Summary statistics of shortest time to positivity, in days, by visit for participants with at least one screening time to positivity value. [file 12879_2022_7250_MOESM1_ESM.docx]

**Additional Tables**

**Table S1. Summary of time to positivity (TTP) counts, by visit**

| **Visit** | **Number of participants with one TTP measurement**  **(1 sputum specimen)** | **Number of participants with two TTP measurements**  **(2 sputum specimens)** | **Number of participants with three TTP measurements**  **(3 sputum specimens)** | **Number of participants contributing at least one measurement** | **Total # of TTP measurements** |
| --- | --- | --- | --- | --- | --- |
| Screening | 13 | 21 | 37 | 71 | 166 |
| Baseline | 12 | 16 | 31 | 59 | 137 |
| Month 1 | 11 | 14 | 28 | 53 | 123 |
| Month 2 | 16 | 9 | 22 | 47 | 100 |
| Month 3 | 9 | 9 | 24 | 42 | 99 |
| Month 4 | 9 | 11 | 28 | 48 | 115 |
| Month 5 | 16 | 9 | 20 | 45 | 94 |
| Month 6 | 13 | 10 | 26 | 49 | 111 |

**Table S2: Repeatability of time to positivity, by visit**

| **Visit** | **Number of participants** | **Intraclass correlation coefficient*** |
| --- | --- | --- |
| Screening | 58 | 0.64 |
| Baseline | 46 | 0.48 |
| Month 1 | 42 | 0.70 |
| Month 2 | 31 | 0.59 |
| Month 3 | 32 | 0.54 |
| Month 4 | 38 | 0.39 |
| Month 5 | 26 | 0.69 |
| Month 6 | 30 | 0.74 |
| *intraclass correlation coefficients of log-transformed time to positivity for all participants with at least two sputum samples at the visit | | |

**Table S3. Summary statistics of shortest time to positivity, in days, by visit for participants with at least one screening time to positivity value**

|  | **Converters** | | **Nonconverters** | | **All participants** | |
| --- | --- | --- | --- | --- | --- | --- |
| **Visit** | **N** | **Median**  **(IQR)** | **N** | **Median**  **(IQR)** | **N** | **Median**  **(IQR)** |
| Screening | 10 | 10.5  (9.4) | 61 | 4.2  (2.8) | 71 | 4.5  (3.6) |
| Baseline | 4 | 6.0  (6.8) | 52 | 4.6  (3.5) | 56 | 4.6  (3.8) |
| Month 1 | 2 | 9.4  (3.1) | 50 | 4.6  (3.8) | 52 | 4.7  (4.5) |
| Month 2 | 1 | 20.5  (N/A) | 45 | 4.6  (5.8) | 46 | 4.7  (6.2) |
| Month 3 | 0 | N/A | 40 | 4.8  (2.5) | 40 | 4.8  (2.5) |
| Month 4 | 2 | 12.1  (13.3) | 44 | 4.5  (3.8) | 46 | 4.6  (4.6) |
| Month 5 | 1 | 19  (N/A) | 40 | 4.9  (6.6) | 41 | 5.0  (6.9) |
| Month 6 | 1 | 22.5  (N/A) | 34 | 4.4  (2.4) | 35 | 4.4  (2.6) |

N represents the number of participants contributing samples per visit.

IQR, inter-quartile range

N/A, not applicable
